# Recovery of Patient-Reported Outcome Measures vs Gait Parameters Obtained by Instrumented Insoles After Tibial and Malleolar Fractures: Prospective Longitudinal Observational Study

**DOI:** 10.2196/71022

**Published:** 2025-06-16

**Authors:** Elke Warmerdam, Marianne Huebner, Caroline Stoll, Andrey Ivanovic Lange, Bergita Ganse

**Affiliations:** 1 Innovative Implant Development (Fracture Healing) Departments and Institutes of Surgery Saarland University Homburg Germany; 2 Department of Statistics Michigan State University East Lansing, MI United States

**Keywords:** fracture, gait parameters, generalized additive model, hypertension, instrumented insoles, lower leg, mixed-effects models, patient-reported outcome measures, PROMIS, wearable

## Abstract

**Background:**

New technologies from the field of mobile health (mHealth) are increasingly used to improve patient monitoring during rehabilitation. While in recent years, mobile phones, health apps, personal digital assistants, and smartwatches opened up new diagnostic and monitoring opportunities for patients, the development of innovative sensor devices, such as instrumented insoles, has now reached a sufficient level of usability with promising opportunities for clinical practice. According to research on the best method for monitoring recovery after musculoskeletal injury or surgery, the Patient-Reported Outcome Measurement Information System (PROMIS) and wearables such as instrumented insoles are among the most promising newer options. However, it is unknown how a patient’s health perception and improvements in instrumented insole-derived gait parameters correlate after surgery for tibial or malleolar fractures.

**Objective:**

This study aimed to compare the longitudinal trajectories in separate PROMIS (sub)scores with gait and further patient-specific parameters, as well as associations between PROMIS scores and gait parameters. It was also aimed to determine the influence of anthropometric parameters and comorbidities.

**Methods:**

A total of 85 patients (39 women and 46 men; average age 50.8, SD 17.1 years) requiring surgery after tibial or malleolar fractures were included in this prospective longitudinal observational study. In the hospital and during follow-up visits, the patients completed the PROMIS Global Health and Pain Interference questionnaires. During the same visits, individually fitted instrumented insoles with 16 pressure sensors, an accelerometer, and a gyroscope each were used to assess the maximal force, pressure distribution, and angular velocity during walking with data being recorded at 100 Hz. Statistical analyses were conducted using linear mixed effect models, pairwise Spearman correlation coefficients, and generalized additive models.

**Results:**

The gait parameters assessed via the instrumented insoles quickly improved during the first 3 months after surgery, followed by a slowing of further improvement. After surgery, the PROMIS scores increased or decreased to extrema that were reached after 6 weeks to 3 months, followed by a return to preinjury values. Between 3 and 6 months, no significant improvements in PROMIS scores were observed. Between 6 months and 1 year, the Physical Health and Mental Health scores still improved significantly (*P*=.003 in both cases). Men had better Physical Health and lower Pain Interference scores than women (*P*=.01 and *P*=.03, respectively). Hypertension had a negative effect on the Physical Health score (*P*=.03). The associations between the PROMIS score and gait parameters were strongest at approximately 3 months after surgery, predominantly between the Pain Interference score and gait parameters.

**Conclusions:**

The patients’ perception improved later than the objective gait parameters obtained by instrumented insoles did. When the gait pattern improved, pain perception correlated with the gait parameters.

**Trial Registration:**

German Clinical Trials Registry DRKS00025108; https://drks.de/search/en/trial/DRKS00025108

## Introduction

Wearable devices such as instrumented insoles have been shown to provide excellent objective measures for monitoring rehabilitation progress after surgery and injury, whereas responses obtained from questionnaires reflect subjective patient perspectives [1-3]. It is currently unknown whether and how the trajectories of these separate measures differ throughout recovery. This information, however, is crucial for drawing correct conclusions from such data in clinical practice and research.

Instrumented insoles are equipped with several pressure sensors and often an inertial measurement unit (IMU) containing triaxial accelerometers and gyroscopes. The total force and pressure distribution curves, as well as the IMU data of the gait cycle, can be used to extract parameters to monitor the progress of healing of lower limb fractures [4,5]. Among the most useful parameters for monitoring fracture healing progress with instrumented insoles are the pressure distribution parameters [6,7]. The greatest improvements in the gait pattern are observed throughout the first 3 months after surgery, with additional, smaller improvements up to at least 6 months [8-10].

In addition, patients’ perspectives obtained in a standardized manner, such as with the Patient-Reported Outcome Measurement Information System (PROMIS), can provide valuable information. Several questionnaires are being increasingly used in orthopedic care [2,11,12]. The Pain Interference and Physical Function subscales are among the most popular PROMIS subscales [11,13]. The Global Health Questionnaire includes a Mental Health component and a Physical Health component and has been used in several studies after fracture treatment [14-18]. In these studies, the Mental and Physical Health scores for patients with lower extremity fractures were worse than those for patients with upper extremity fractures [15,18]. Mental and Physical Health scores are also associated with fracture severity in patients with malleolar fractures [16]. The Mental Health score was correlated with the Physical Function score [14].

In studies in which the PROMIS (Physical Health or Physical Function) was completed more than twice after surgery with a follow-up time of more than 1 year, improvements in the Physical Function score were observed up to 6 months [19]. Improvements in the Physical Health score were observed up to 1 year after surgery [15]. The PROMIS, which measures mobility and physical function, is correlated with the RAND-36 Physical Function Scale, the Short Musculoskeletal Function Assessment, the Foot and Ankle Ability Measures, and the University of California Los Angeles Activity Scale [20]. However, the associations of the PROMIS Global Health and Pain Interference scores after surgery remain largely unknown. Therefore, it is unclear whether longitudinally collected patient-reported outcome data after fractures can inform health care providers and how these data can be compared with data obtained from instrumented insoles.

To answer these questions, the primary hypothesis of this study was that the longitudinal trajectories of the PROMIS scores for Physical Health, Mental Health, and Pain Interference differ from those of gait parameters assessed with instrumented insoles among patients with tibial and malleolar fractures, with sex, age, fracture type, implant type, BMI, diabetes, hypertension, cancer, and previous injuries as explanatory variables. The secondary hypothesis was that PROMIS scores are associated with changes in gait parameters of the injured leg after surgery.

## Methods

### Participants

Patients who underwent surgery for tibial and malleolar fractures at Saarland University Hospital, Germany, were prospectively recruited between February 2022 and May 2024. The exclusion criteria were being younger than 18 years, inability to give consent, requiring walking aids before the fracture, other injuries, or disorders that affect mobility and pregnancy. Patients were recruited during their hospital stay after surgery. The study size was determined by the length of the recruitment period and not based on a sample size calculation. The length of the recruitment period was determined by the ending nonpermanent work contracts of the staff.

### Data Collection

During the hospital stay, anthropometric variables, such as sex, age, weight, and height, as well as information about comorbidities, previous injuries, and smoking status, were recorded. Details on the fracture and implant types (nail or plate) were extracted from the electronic hospital system.

During each follow-up visit, patients were asked to fill out the PROMIS Global Health questionnaire (version 1.2) and the Pain Interference short form 8a questionnaire (Item Bank version 1.0), which were developed for adults [21]. The paper-based questionnaires were written in German and completed by each patient without assistance. The baseline PROMIS forms were collected during the hospital stay after surgery, at which time patients were asked to assess their preinjury condition. Patients then returned for outpatient follow-up visits 6 weeks, 3 months, 6 months, 9 months, and 1 year after surgery. Each time, they completed the same questionnaires to document their current condition.

Gait assessments were performed with instrumented insoles containing 16 pressure sensors, a triaxial accelerometer, and a triaxial gyroscope (OpenGO insoles, Moticon GmbH). The insoles were fitted to the shoe soles of each patient, and then calibrated to their body weight. Data were recorded on a 10-m walkway during regular gait at each patient’s own preferred speed at 100 Hz. Gait assessments took place during the initial hospital stay and during each outpatient follow-up visit together with the PROMIS assessments.

### Data Processing

The PROMIS Global Health items were used to calculate the raw Global Physical Health score and the Global Mental Health score. The raw scores of the Global Physical Health, Global Mental Health, and Pain Interference scores were used to calculate T scores for further analysis [22]. If there were missing responses to one specific score, only this score was excluded for that visit of the patient.

**Figure 1:**
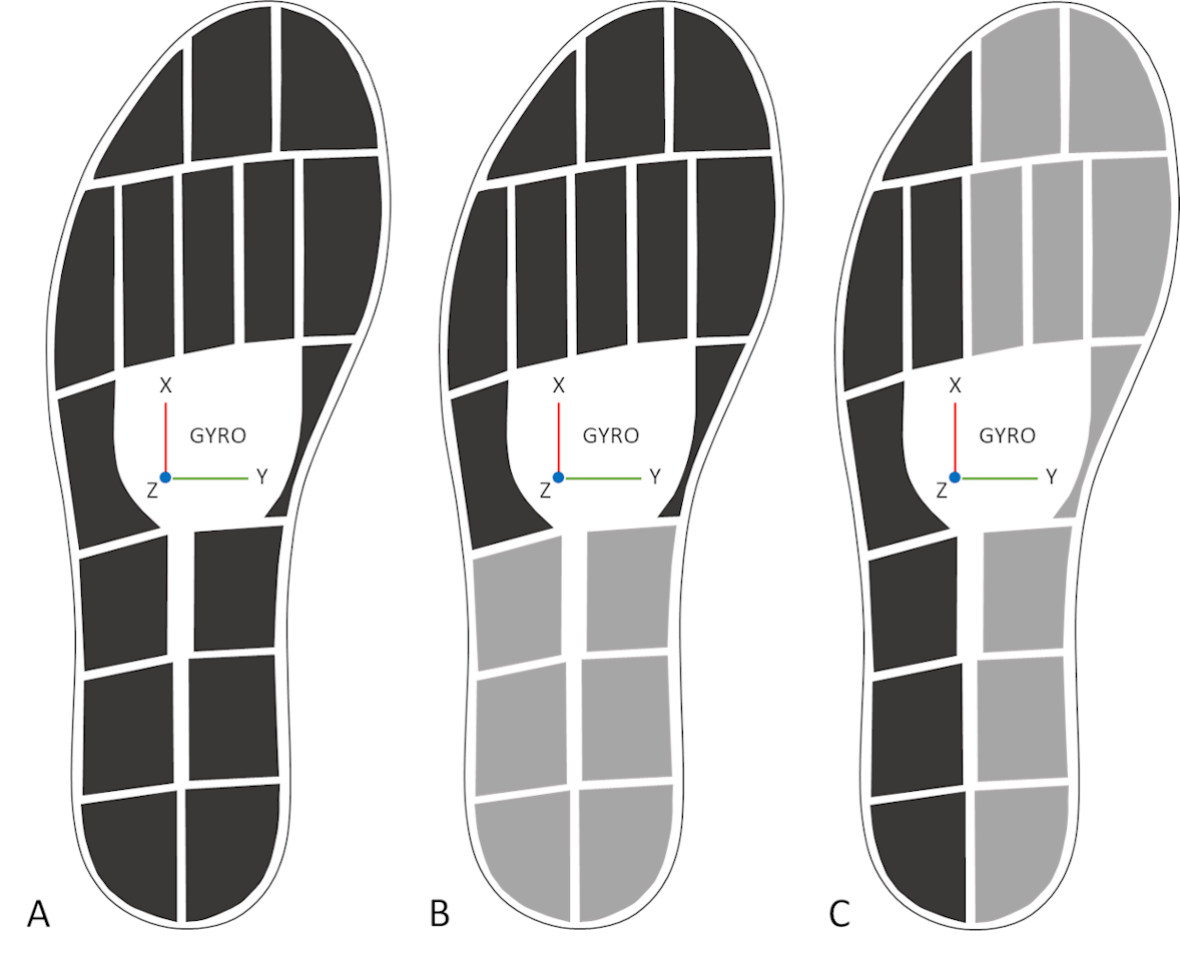
Layout of pressure sensors and location and orientation of the gyroscope in the instrumented insole. (A). Total force, which is calculated from all the pressure sensor data pooled. (B). Forefoot (dark gray) and hindfoot (light gray) pressure sensor combination. (C). Lateral (dark gray) and medial (light gray) pressure sensor combinations.

The insole software (OpenGO, Moticon GmbH) provided the total vertical force, the single pressure sensor data, and the gyroscope data (Figure 1A). A custom-written MATLAB (The MathWorks, Inc) code was used to process the data further. To remove noise from the data, a fourth-order Butterworth filter with a cutoff frequency of 6 Hz was applied to all the data. The detection of the initial and final contact of a stride was based on an arbitrary threshold of 30 N in the vertical force data. The first instant at which the force exceeded the threshold and remained above this threshold for at least 300 milliseconds was defined as initial contact. The first instant after the initial contact when the force dropped under 30 N was defined as the final contact. The force data were normalized by first dividing them by the body weight (in Newtons) and then multiplying them by 100 to obtain the force data as a percentage of body weight. The pressure data were normalized to the percentage of body weight and then divided by the number of pressure sensors that were combined to obtain the pressure underneath the forefoot, hindfoot, lateral, and medial sides of the foot (Figures 1B and 1C). Only the angular velocity around the mediolateral axis was used for further analysis. From the total force, the 4 types of pressure data and the angular velocity data, the maximum per stride of the injured side were extracted. The first and last few strides were discarded, such that only the 5 strides in the middle part of the 10-m walkway were kept for analysis to minimize the effects of gait initiation and termination. The average of these 5 strides on the injured side was used for analysis.

Whether patients experienced fracture union or nonunion was judged on the basis of radiographs taken 6 months after surgery as part of the regular clinical care procedure. Nonunion was defined as a lack of visible callus bridging on radiographs and was determined by an experienced specialist orthopedic surgeon. The lack of visible callus bridging was chosen as an objective criterion to avoid subjective bias. Similarly, the questionnaires and treadmill recordings were objective measures.

### Statistical Analysis

Continuous variables are summarized with means and SD, and categorical variables are summarized with frequencies and percentages in the tables. Wilcoxon or Pearson tests were used to compare these variables between males and females, as appropriate.

Disjoint time intervals were defined as 0-15 days (preinjury level for PROMIS and first week for gait analysis), 16-55 days (6 weeks), 56-110 days (3 months), 111-200 days (6 months), 201-300 days (9 months), and more than 300 days (1 year). If more than 1 measurement occurred during the same time interval for a particular patient, only the last measurement was included in the analysis set for that patient.

A 5-point score change in the T scores of the PROMIS Physical Health, PROMIS Mental Health, and PROMIS Pain Interference between visits was considered clinically meaningful. This corresponds to a moderate effect size change of 0.5 SD, as shown in other studies with orthopedic patient populations [23,24].

Linear mixed effects models (LMMs) were used to estimate the time course of the PROMIS components (physical health, mental health, and pain interference) after surgery. Random effects accounted for variation at the individual level. Patient data from all laboratory visits (up to 6 visits) were used for the estimation. Separate models were developed for each of the 3 PROMIS constructs. The explanatory variables were sex, age, fracture type, implant type, BMI, diabetes, hypertension, cancer, and previous injuries. These variables were included in a full model, and the variables with the largest coefficients (absolute size larger than 1) were included in all final models (sex and hypertension). LMMs were also used to estimate the time course of gait parameters (total force, forefoot, hindfoot, lateral, and medial pressure, and angular velocity) after surgery in a similar fashion as the PROMIS.

Pairwise Spearman correlation coefficients were calculated between PROMIS components (physical health, mental health, and pain interference) and gait parameters (maximal force, medial, lateral, forefoot, hindfoot pressure, and angular velocity) at 3 time points, 6 weeks, 3 months, and 6 months, since during these time points, the largest changes in both PROMIS and gait parameters were expected.

Generalized additive models for location, scale, and shape (GAMLSS) [25] were used to estimate the distribution of gait parameters on the injured leg for a given score in the PROMIS Physical Health, Mental Health, and Pain Interference (Model 1) below. Separate models were constructed for each gait parameter (maximal force, medial, lateral, forefoot, hindfoot, and angular velocity). GAMLSSs are regression analyses that allow modeling of the outcome with a parametric distribution whose moments are estimated as smooth curves for the covariate PROMIS scores. We used the Box-Cox Cole Green (BCCG) distribution for the outcome [26]. The moments for these distributions correspond to the median, coefficient of variation, and Box-Cox power transformation needed to adjust for skewness in the distribution. The associations with the gait parameters were estimated using penalized B-splines for each PROMIS subscore.

Model 1:

µ = a_µ_ + pb(Physical Health) + pb(Mental health) + pb(Pain Interference)

log(*σ*) = *a_σ_* + pb(Pain Interference)

*v* = *a_v_*

All analyses were conducted using R (version 4.0.0; R Core Team) [27]. The lmerTest package was used for the mixed effects models. The GAMLSS package was used to estimate the associations of PROMIS scores with the gait parameters of the injured legs. *P* values of <.05 indicate significance in all tests.

### Ethical Considerations

The study protocol was approved by the institutional review board of Saarland Medical Board (Ärztekammer des Saarlandes, Germany, application number 30/21). Written informed consent was obtained from all participants before the start of the measurements. Participants had the opportunity to opt out at any time without needing to give reasons. This observational, prospective longitudinal cohort study was conducted in accordance with the Declaration of Helsinki. The data were pseudonymized. The participants did not receive any financial or nonfinancial compensation for their study participation.

## Results

### Overview

**Figure 2:**
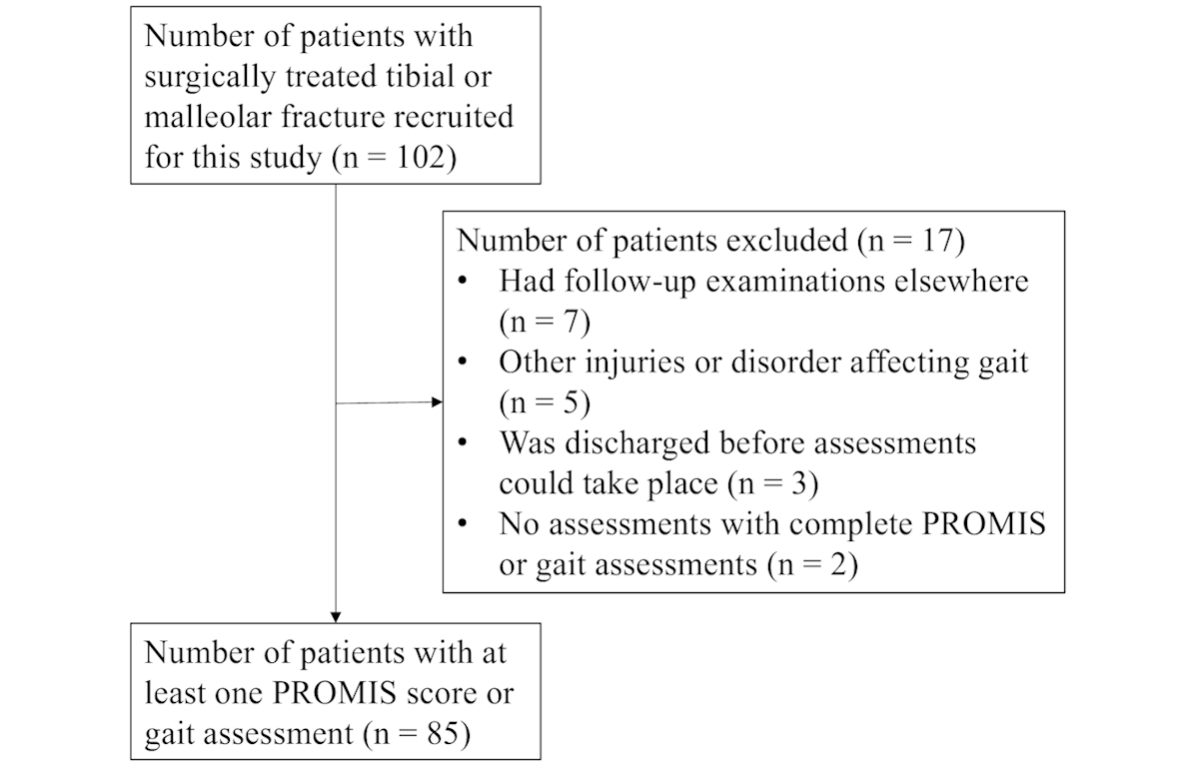
Flowchart of patients included in this study. PROMIS: Patient-Reported Outcome Measurement Information System.

A total of 102 patients were initially recruited. Out of this dataset, patients were excluded in the case of the inability to assess gait or PROMIS (missingness of observational units). Accordingly, 85 patients (39 women and 46 men) were included in the analysis dataset (Figure 2). Smoking and diabetes were more prevalent in male patients than in female patients (Table 1). Age, comorbidities, fracture type, implant type, and previous injuries did not differ between men and women. There were no sex differences in the PROMIS scores at the preinjury level or at 6 months or 1 year after surgery (Table 2).

**Table 1:** Demographics and diagnoses by sex.

Demographics^a^			Total (N)		Female (n=39)		Male (n=46)		Combined (n=85)		Test statistics				
											F test (*df*)		Chi-square (*df*)		*P* value
Age (years), mean (SD)			85		52.8 (17.9)		49.0 (16.4)		50.8 (17.1)		1.11 (1, 83)		—^b^		.29
BMI (kg/m^2^), mean (SD)			85		26.43 (4.89)		27.35 (3.47)		26.93 (4.18)		1.80 (1, 83)		—		.18
Implant type: n nail (%)			85		5 (13)		12 (26)		17 (20)		—		2.32 (1)		.13
Smoking, n (%)			84		3 (8)		16 (35)		19 (23)		—		8.60 (1)		.003
Hypertension, n (%)			85		6 (15)		7 (15)		13 (15)		—		0 (1)		.98
Diabetes, n (%)			85		1 (3)		7 (15)		8 (9)		—		3.96 (1)		.05
Cancer, n (%)			85		2 (5)		0 (0)		2 (2)		—		2.42 (1)		.12
Previous injuries, n (%)			85		6 (15)		7 (15)		13 (15)		—		0 (1)		.98
**Fracture type, n (%)**															
	Proximal tibia	85		10 (26)		10 (22)		20 (24)		—		4.21 (2)		.12	
	Tibial shaft	—		6 (15)		16 (35)		22 (26)		—		—		—	
	Malleolar	—		23 (59)		20 (44)		43 (51)		—		—		—	

^a^For continuous variables, means (SDs) and *F* test scores of the Wilcoxon tests are reported. For categorical variables, n (percentages) and chi-square values from Pearson tests are reported.

^b^Not applicable.

**Table 2:** Patient-Reported Outcome Measurement Information System scores preinjury and 6 months and 1 year after surgery, separated by sex.

PROMIS system		Total (N)	Female, mean (SD)	Male, mean (SD)	Combined, mean (SD)	Test statistics	
						*F* test (*df*)	*P* value
**PROMIS^a^ T score preinjury level**							
	Physical Health	70	49.60 (10.25)	51.68 (7.39)	50.90 (8.82)	0.43 (1, 67)	.51
	Mental Health	69	50.91 (10.53)	51.24 (9.22)	51.21 (9.72)	0.02 (1, 66)	.90
	Pain Interference	51	55.0 (13.6)	48.3 (10.3)	51.2 (12.1)	3.20 (1, 48)	.08
**PROMIS T score 6 months after surgery**							
	Physical Health	39	41.84 (6.30)	44.45 (6)	43.33 (6.12)	2.47 (1, 36)	.13
	Mental Health	40	44.36 (8.36)	45.82 (7.30)	45.16 (7.64)	0.22 (1, 37)	.65
	Pain Interference	26	57.02 (7.24)	58.08 (8.15)	57.62 (7.43)	0.14 (1, 23)	.71
**PROMIS T score 1 year after surgery**							
	Physical Health	13	46.0 (8.4)	54.7 (10.0)	49.3 (9.7)	3.05 (1, 11)	.11
	Mental Health	13	48.6 (12.2)	54.4 (11.5)	50.9 (11.8)	0.91 (1, 11)	.36
	Pain Interference	10	54.35 (11.53)	53.15 (9.84)	53.87 (10.32)	0.09 (1, 8)	.77

^a^PROMIS: Patient-Reported Outcome Measurement Information System.

### PROMIS Trajectories Throughout the Healing Process

**Figure 3:**
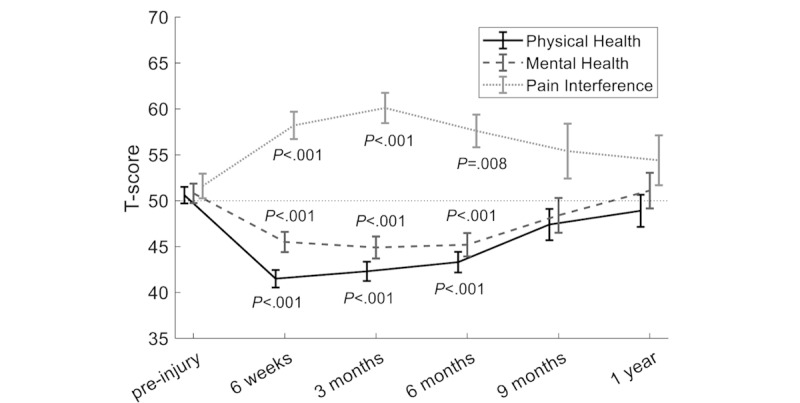
The trajectories of the Patient-Reported Outcome Measurement Information System scores and the means with SE are presented. A decrease in physical health and mental health and an increase in pain interference indicate a worse score.

After surgery, the Physical Health, Mental Health and Pain Interference (PROMIS) scores increased or decreased to extrema that were reached after 6 weeks to 3 months, followed by a return to preinjury values (Figure 3). All scores were significantly worse than they were before surgery during the first 6 months after surgery (Table S1 in Multimedia Appendix 1). No significant differences were found between preinjury, 9 months, and 1 year after surgery. Between 3 and 6 months, no significant improvements in PROMIS scores were observed. Between 6 months and 1 year, the Physical Health and Mental Health scores improved significantly (*P*=.003 in both cases). Compared with women, men had better Physical Health and lower Pain Interference scores (*P*=.01, *P*=.03, respectively). Hypertension had a negative effect on the Physical Health score (*P*=.04, Table S1 in Multimedia Appendix 1).

### Gait parameter Trajectories Throughout the Healing Process

**Figure 4:**
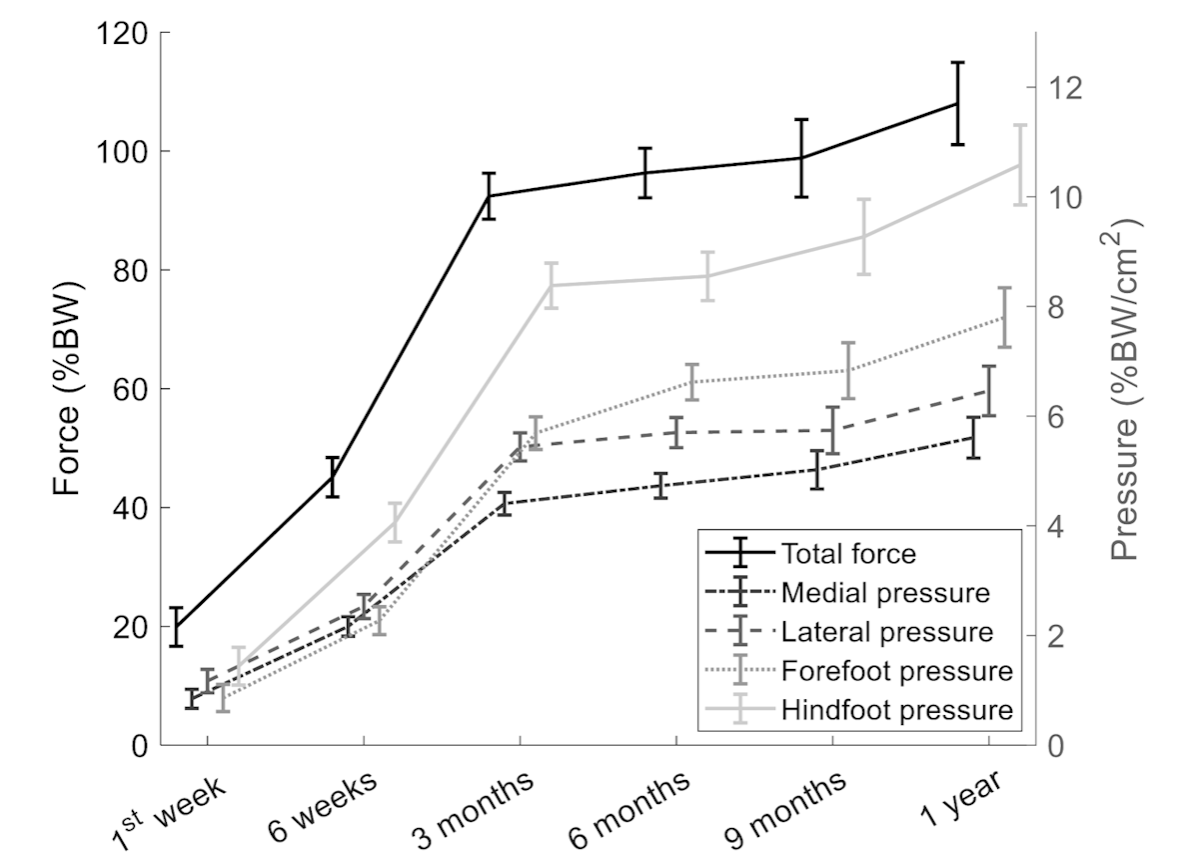
Trajectories of insole-derived gait parameters’ means with SE. All the parameters at all the time points were significantly different from those at the first week. BW: body weight.

Compared with the first assessment shortly after surgery, all gait parameters were significantly better at all follow-up assessments (Figure 4). After an initial fast improvement, no significant difference was found between 3 and 6 months for any of the gait parameters, except for the improvement seen in forefoot pressure. After 6 months, there was no further improvement in total force or lateral or forefoot pressure, whereas the medial and hindfoot pressures continued to improve significantly for another 6 months. Neither sex nor hypertension affected gait parameters (Table S2 in Multimedia Appendix 1).

### Associations Between the PROMIS Score and Gait

The associations between the PROMIS score and gait parameters were calculated for patients whose data were collected at the same time intervals. At 6 weeks, the correlation coefficients of the PROMIS scores and gait parameters were small (absolute values less than 0.3). The largest correlation coefficients (defined as absolute correlation coefficients close to 0.5) between all PROMIS scores and gait parameters except for the maximal total force were observed at 3 months. At 6 months, the corresponding correlation coefficients were again smaller (Table 3).

**Table 3:** Correlation coefficients (Spearman) of Patient-Reported Outcome Measurement Information System scores with gait parameters (n is due to missingness of either gait or patient-reported outcome measurement information system).

Time post surgery		Total force	Medial pressure	Lateral pressure	Forefoot pressure	Hindfoot pressure	Angular velocity
**PROMIS^a^ Physical Health**							
	6 weeks (n=37)	0.200	0.101	0.246	0.054	0.237	0.153
	3 months (n=28)	0.257	0.379	0.452	0.282	0.477	0.453
	6 months (n=26)	0.301	0.247	0.407	0.336	0.373	0.454
**PROMIS Mental Health**							
	6 weeks (n=37)	0.246	0.218	0.291	0.194	0.268	0.241
	3 months (n=28)	0.252	0.422	0.526	0.461	0.486	0.470
	6 months (n=26)	0.253	0.184	0.372	0.264	0.297	0.478
**PROMIS Pain Interference**							
	6 weeks (n=37)	–0.124	–0.057	–0.192	–0.022	–0.155	–0.065
	3 months (n=28)	–0.328	–0.550	–0.538	–0.437	–0.513	–0.550
	6 months (n=26)	–0.368	–0.410	–0.456	–0.466	–0.382	–0.545

^a^PROMIS: Patient-Reported Outcome Measurement Information System.

Generalized additive models [25] were used to estimate the associations of PROMIS scores (Mental Health and Physical Health, and Pain Interference) with each gait parameter on the injured leg for a given score on the PROMIS at 3 months (Table 4). This time point was chosen because of the greater correlations between PROMIS scores and gait parameters and because of the larger sample size than those at later time points. Among the PROMIS subscores, the mean pain interference score was significantly associated with all gait parameters. Physical health was associated with medial and forefoot pressure. Mental health was not associated with gait data.

**Table 4:** Coefficients of generalized additive models for location, scale, and shape for models with Patient-Reported Outcome Measurement Information System scores as explanatory variables and each gait parameter as the dependent variable at 3 months (n=28). The estimates (SE) and *P* values are presented.

		Dependent variable: gait parameters																									
		Total force				Medial pressure				Lateral pressure					Forefoot pressure					Hindfoot pressure					Angular velocity		
		Mean (SD)		*P* value		Mean (SD)		*P* value		Mean (SD)		*P* value		Mean (SD)			*P* value		Mean (SD)			*P* value		Mean (SD)			*P* value
**Mu link function: identity**																											
	Intercept	365.46 (86.91)		<.001		21.55 (2.03)		.001		17.04 (5.87)		.009		22.97 (2.95)			<.001		21.37 (5.48)			<.001		656.65 (245.55)			.01
	Physical Health	–2.14 (1.89)		.27		–0.15 (0.05)		.004		–0.03 (0.08)		.70		–0.37 (0.10)			.001		0.04 (0.10)			.72		–2.82 (2.91)			.34
	Mental Health	0.96 (1.07)		.38		0.03 (0.03)		.29		0.02 (0.04)		.64		0.13 (0.07)			.06		0.05 (0.04)			.26		3.12 (2.41)			.21
	Pain Interference	–3.83 (0.79)		<.001		–0.20 (0.02)		<.001		–0.19 (0.08)		.02		–0.096 (0.02)			<.001		–0.30 (0.07)			<.001		–5.87 (2.45)			.03
**Sigma link function: log**																											
	Intercept	–7.08 (2.74)		.02		–9.77 (2.17)		<.001		–5.49 (3.98)		.18		–10.59 (2.01)			<.001		–8.85 (5.33)			.11		–3.83 (1.58)			.02
	Pain Interference	0.10 (0.05)		.05		0.14 (0.04)		.001		0.08 (0.07)		.30		0.16 (0.04)			<.001		0.14 (0.10)			.19		0.04 (0.03)			.19
**Nu link function: identity**																											
	Intercept	1.20 (0.52)	.03		1.75 (0.55)		.005		1.51 (0.55)		.01		1.17 (0.39)			.007		1.80 (0.59)			.006		1.40 (1.82)			.45	

## Discussion

### Principal Findings

The PROMIS scores and gait parameters revealed distinct trajectories throughout the healing process of the lower leg and malleolar fractures. The gait parameters showed the most pronounced improvements during the first 3 months, whereas the PROMIS scores lagged and showed worsening to an extremum, followed by improvements at approximately 6 to 9 months. Nen had better Physical Health and lower Pain Interference scores than women. Hypertension had a negative effect on the Physical Health score.

#### PROMIS

The Mental Health and Pain Interference scores were at their worst 3 months after surgery, indicating that, according to the patient’s perspective, it takes longer for their health to improve than indicated by the insole gait measurements. Patients might have expected to be free of symptoms at this time and may be disappointed about their slower-than-expected progress. Similar psychological effects are known from populations in confined environments, such as when over-wintering on Antarctic stations, where the worst mood, called the third-quarter phenomenon, is usually reported after 3 quarters of the mission length [28]. Unmet expectations have been shown to be a mental burden for patients [29]. Different patient experiences and recovery challenges have been reported after ankle fracture surgery, with the key themes of “understanding the recovery journey,” “navigating the healthcare system,” “understanding personal physical capabilities,” “building confidence for weight-bearing,” and “resuming daily activities” [30]. In addition, mobility, loss of independence, health care, psychological effects, social and family life, ankle symptoms, sleep disturbance and fatigue, and activities of daily living have been identified as majorly relevant factors for patients with ankle fractures [31]. These patients considered 5 factors to be particularly important: ability to regain independence, sleep quality and quantity, ability to drive, ability to walk without walking aids or weight-bearing restrictions, and radiological union [31]. The worst PROMIS scores 3 months after surgery in this study may therefore likely be explained by the persistence of at least some of these factors. Thus, in future studies, researchers should try to identify the detailed underlying reasons to determine whether interventions may be available to address at least some of them.

#### Correlations Between Gait and PROMIS

In this study, a novel statistical approach was used to model the associations of PROMIS scores with gait parameters. The new approach considers that the variability of a variable across a range of measured values can change (heteroscedasticity) and can model complex distributions. The greatest correlations were found approximately 3 months after surgery. These values were between those of pain interference and gait parameters, indicating that the amount of pain is associated with gait patterns. This finding aligns with a study on hip arthroscopy, where moderate correlations were found 3 months after surgery between gait speed and the PROMIS Physical Function and Pain scores [32]. In other patient groups with chronic pain conditions, such as knee osteoarthritis, patellofemoral pain, low back pain, and neck pain, patients walk more slowly, with shorter strides, and smaller joint angles and moments [33-36]. Therefore, pain and gait seem to be associated with each other. Pain reliever use and partial weight bearing may be the cause of the lack of significant correlations between gait and pain interference 6 weeks after surgery.

According to the generalized additive models, PROMIS Mental Health scores were not associated with gait 3 months after surgery. In people with mental health problems but without physical problems, mental health is associated with gait [37,38]. It is possible that in our cohort, mental health also influenced gait parameters, but the accompanying physical health problems might have had a greater effect on gait.

The trajectories of PROMIS scores and gait parameters can be used to inform patients what they can expect after their surgery. This might provide them with a better understanding of how quickly different health aspects improve over time. In the patients with nonunion analyzed in the present study (n=7), the PROMIS score, and mainly the Pain Interference score, returned to preinjury levels more slowly between 6 months and one year after surgery.

#### Effects of Sex and Hypertension

In this study, men reported better physical health and lower pain interference than women. Similar sex differences were found in PROMIS scores of patients with joint arthroplasty [39] and populations with lupus erythematosus [40], neuromuscular disease, multiple sclerosis, postpolio syndrome, or spinal cord injury [41]. This finding is therefore in line with the literature and not specifically related to this particular patient collective.

Arterial hypertension had a negative effect on the Physical Health score. This finding is certainly related to the fact that arterial hypertension is usually associated with further comorbidities of the metabolic syndrome, including obesity, insulin resistance and diabetes, dyslipidemia [42], as well as in women with polycystic ovarian syndrome [43]. Similar to the sex difference found in the PROMIS results of this study, the finding that arterial hypertension was associated with a lower Physical Health score is not surprising.

#### Implications for Clinical Practice

In this study, the PROMIS trajectories provided information about the healing process of ankle or tibial fractures. However, the usability of these subjective measures to monitor fracture healing might be limited because of the potential influence of secondary gain. In clinical practice, patients may score questionnaires worse than they actually perceive them to receive longer paid sick leave or further rehabilitation prescriptions [44]. Since long-term limitations after work accidents in some countries result in permanent payments of disability pensions [45], to avoid abuse, the decision of whether problems persist must not only depend on questionnaire data but also apply more objective outcomes. Since it was clear to the patients in this study that the data were used for research only and that they had no effect on their treatment or potential secondary benefit, we expect that secondary gain played no or only a minor role in this study. However, the results from questionnaires in patients with injuries need to be handled with care and, if indicated, combined with more objective measures to avoid abuse. Among such more objective measures, gait analyses certainly play an important role. Various technologies and products exist to perform gait analyses in patients with fracture [4].

#### Impact on the Field of Mobile Health

While the development of instrumented insoles has only recently reached a level that is suitable for daily use in a clinical environment, this study demonstrates a new benefit of this technology. The same data could also have been recorded with a ground-based force plate. However, insoles are inexpensive and with them, gait data can be recorded in any outpatient clinic or private practice, just as in settings where less financial resources are available. Another benefit of instrumented insoles over laboratory-based measurements is that they allow continuous monitoring of the daily lives of patients [5]. As patients after injury are initially unable to walk on a treadmill, especially if they still use walking aids, instrumented treadmills are less suitable for this purpose. Other than peak forces and acceleration data, the use of ground-reaction force-derived parameters recorded with instrumented insoles is a recent development with basic studies on the nature of the ground-reaction force curve obtained via insoles only having been reported recently [46-48]. The clinical usability to monitor and predict recovery based on these parameters has been demonstrated in several studies [4,5]. Instrumented insoles can also be used to provide immediate patient feedback on the weight bearing [49]. This study showed a potential benefit of this new technology for rehabilitation monitoring that should be followed up with more studies that compare traditional monitoring methods with instrumented insole monitoring. This way, the true clinical value of this new mobile health (mHealth) technology for clinical practice can be determined. It needs to be pointed out, that the development and evaluation of parameters derived from the raw data is of crucial importance. To be able to conduct this parameter development, large data sets from many patients are needed. Similar parameters may be derived from the data obtained by smart shoes and socks [50,51], which are further new wearable mHealth devices with great potential.

### Strengths and Limitations

A limitation of this study is the incompleteness of the longitudinal data. The reason is that some patients did not return for follow-up visits because they either switched to a local orthopedic surgeon, had no or minor symptoms remaining (3 months after surgery), or were lost to follow-up for unknown reasons. This led to a reduction in sample size over time and a small sample size at 1 year post surgery. Another weakness is that countless gait parameters exist that have not been measured or analyzed in this study. The findings of this study are of course only valid for the few parameters reported. A strength of this study is that the follow-up time of both the PROMIS and gait measurements was one year. The PROMIS scores indicate that it takes up to a year for the scores to return to normal values. If patients who improved did not return, this may have led to an underestimation of the healing progression in the trajectories in our study.

Different types of fractures were pooled: proximal tibial, tibial shaft, distal tibial, and malleolar fractures. This is a limitation, but the authors expect only minor differences in gait and PROMIS between the different fracture types.

### Conclusion

The gait parameters improved during the first 3 months post surgery, whereas the improvements in PROMIS scores lagged, with meaningful differences observed at approximately 6 to 9 months. Thus, the patients’ perceptions improved later than their objective parameters assessed by instrumented insoles did. Pain perception was associated with the gait parameters, but only if they had already improved. Compared with women, men had better physical health and lower pain interference scores. Hypertension had a negative effect on the physical health score.

## Appendix

Multimedia Appendix 1 Additional tables.

## Abbreviations

BCCG: Box-Cox Cole Green
GAMLSS: generalized additive models for location, scale, and shape
IMU: inertial measurement unit
LMM: linear mixed-effects model
mHealth: mobile health
PROMIS: Patient-Reported Outcome Measurement Information System
