# Supplementary material for: Recovery of Patient-Reported Outcome Measures vs Gait Parameters Obtained by Instrumented Insoles After Tibial and Malleolar Fractures: Prospective Longitudinal Observational Study
Source: JMIR Mhealth Uhealth. 2025 Jun 16;13:e71022. doi: 10.2196/71022 (PMC12209729; doi:10.2196/71022)
Supplement: Multimedia Appendix 1 [file mhealth_v13i1e71022_app1.docx]

**Multimedia Appendix 1**

Supplementary Table 1. Mixed effect models of PROMIS scores. The estimate (standard error) and p values are presented.

| Variable | Physical health | Mental health | Pain interference |
| --- | --- | --- | --- |
| Sex | **3.97 (1.58), 0.01** | 3.42 (2.09), 0.11 | **-5.54 (2.47), 0.03** |
| Hypertension | **-4.40 (2.08), 0.04** | -4.22 (2.74), 0.13 | 1.98 (3.20), 0.54 |
| Preinjury vs. 6 weeks | **-8-87 (1.01), <0.001** | **-5.13 (1.10), <0.001** | **6.02 (1.72), <0.001** |
| Preinjury vs. 3 months | **-7.85 (1.08), <0.001** | **-5.54 (1.17), <0.001** | **8.01 (1.87), <0.001** |
| Preinjury vs. 6 months | **-6.83 (1.16), <0.001** | **-5.27 (1.25), <0.001** | **5.42 (2.01), 0.008** |
| Preinjury vs. 9 months | -2.84 (1.80), 0.12 | -1.32 (1.96), 0.50 | 2.96 (3.36), 0.38 |
| Preinjury vs. > 1 year | -1.22 (1.77), 0.49 | 0.67 (1.93), 0.73 | 1.96 (2.93), 0.51 |
| **Contrasts** | | | |
| 3 vs. 6 months | -0.94 (1.22), 0.44 | -0.33 (1.31), 0.81 | 2.45 (2.08), 0.24 |
| 6 months vs. 1 year | **-5.61 (1.84), 0.003** | **-5.86 (1.97), 0.003** | 3.29 (2.99), 0.27 |

Supplementary Table 2. Mixed effect models of gait parameters. The estimate (standard error) and p values are presented.

| Variable | Total force | Medial | Lateral | Forefoot | Hindfoot |
| --- | --- | --- | --- | --- | --- |
| Sex | -5.01 (5.56), 0.370 | -0.17 (0.29), 0.553 | -0.26 (0.33), 0.424 | -0.55 (0.38), 0.154 | 0.58 (0.59), 0.321 |
| Hypertension | 2.99 (7.21), 0.680 | 0.20 (0.38), 0.601 | -0.76 (0.49), 0.126 | 0.09 (0.50), 0.863 | 0.20 (0.76), 0.789 |
| 1^st^ week vs. 6 weeks | **29.26 (4.82), <0.001** | **1.48 (0.26), <0.001** | **1.73 (0.31), <0.001** | **1.78 (0.37), <0.001** | **3.15 (0.50), <0.001** |
| 1^st^ week vs. 3 months | **75.91 (5.12), <0.001** | **3.75 (0.28), <0.001** | **4.42 (0.33), <0.001** | **5.25 (0.40), <0.001** | **7.15 (0.53), <0.001** |
| 1^st^ week vs. 6 months | **77.43 (5.32), <0.001** | **3.95 (0.29), <0.001** | **4.70 (0.34), <0.001** | **5.97 (0.41), <0.001** | **7.30 (0.56), <0.001** |
| 1^st^ week vs. 9 months | **81.25 (8.25), <0.001** | **4.34 (0.44), <0.001** | **4.62 (0.53), <0.001** | **6.44 (0.63), <0.001** | **8.25 (0.86), <0.001** |
| 1^st^ week vs. 1 year | **93.46 (8.71), <0.001** | **5.04 (0.47), <0.001** | **5.44 (0.56), <0.001** | **7.33 (0.67), <0.001** | **9.66 (0.91), <0.001** |
| **Contrasts** | | | |  |  |
| 3 months vs. 6 months | -3.94 (5.12), 0.443 | -0.33 (0.28) 0.240 | -0.26 (0.33), 0.424 | **-2.11 (0.59), <0.001** | -0.17 (0.53), 0.747 |
| 3 months vs. 1 year | **-15.62 (7.53), 0.039** | **-1.20 (0.41), 0.003** | **-1.02 (0.49), 0.037** | **-2.11 (0.59), <0.001** | **-2.20 (0.79), 0.006** |
